# Supplementary material for: Labeling of Bruton’s Tyrosine Kinase (BTK) Inhibitor [11C]BIO-2008846 in Three Different Positions and Measurement in NHP Using PET
Source: Int J Mol Sci. 2024 Jul 18;25(14):7870. doi: 10.3390/ijms25147870 (PMC11277166; doi:10.3390/ijms25147870)
Supplement: Supplementary file 1 [file ijms-25-07870-s001.zip › ijms-3065456-supplementary.pdf]

S1: Analytical Test Summary
BIO-2008846

^{1}H-NMR:

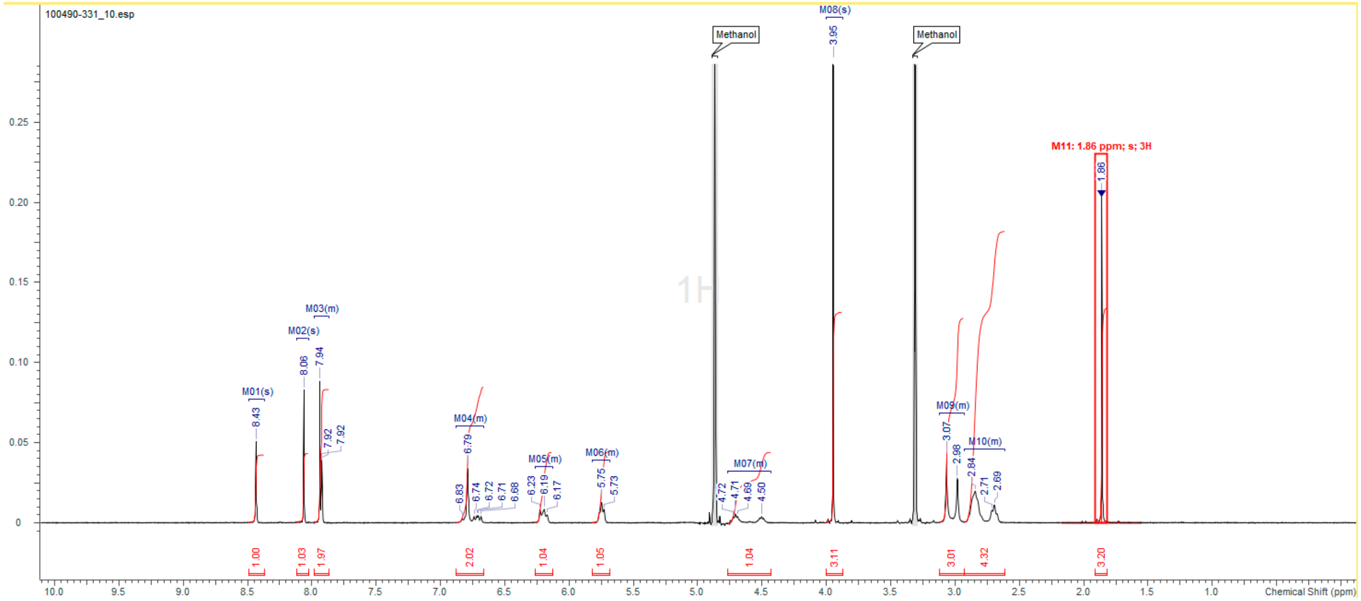

^{1}H NMR (500 MHz, METHANOL-d$_4$) δ 8.43 (s, 1H), 8.06 (s, 1H), 7.89-7.96 (m, 2H), 6.66-6.87 (m, 2H), 6.16-6.27 (m, 1H), 5.68-5.80 (m, 1H), 4.44-4.80 (m, 1H), 3.95 (s, 3H), 2.95-3.10 (m, 3H), 2.64-2.92 (m, 4H), 1.86 (s, 3H)

HPLC–purity, acidic conditions:

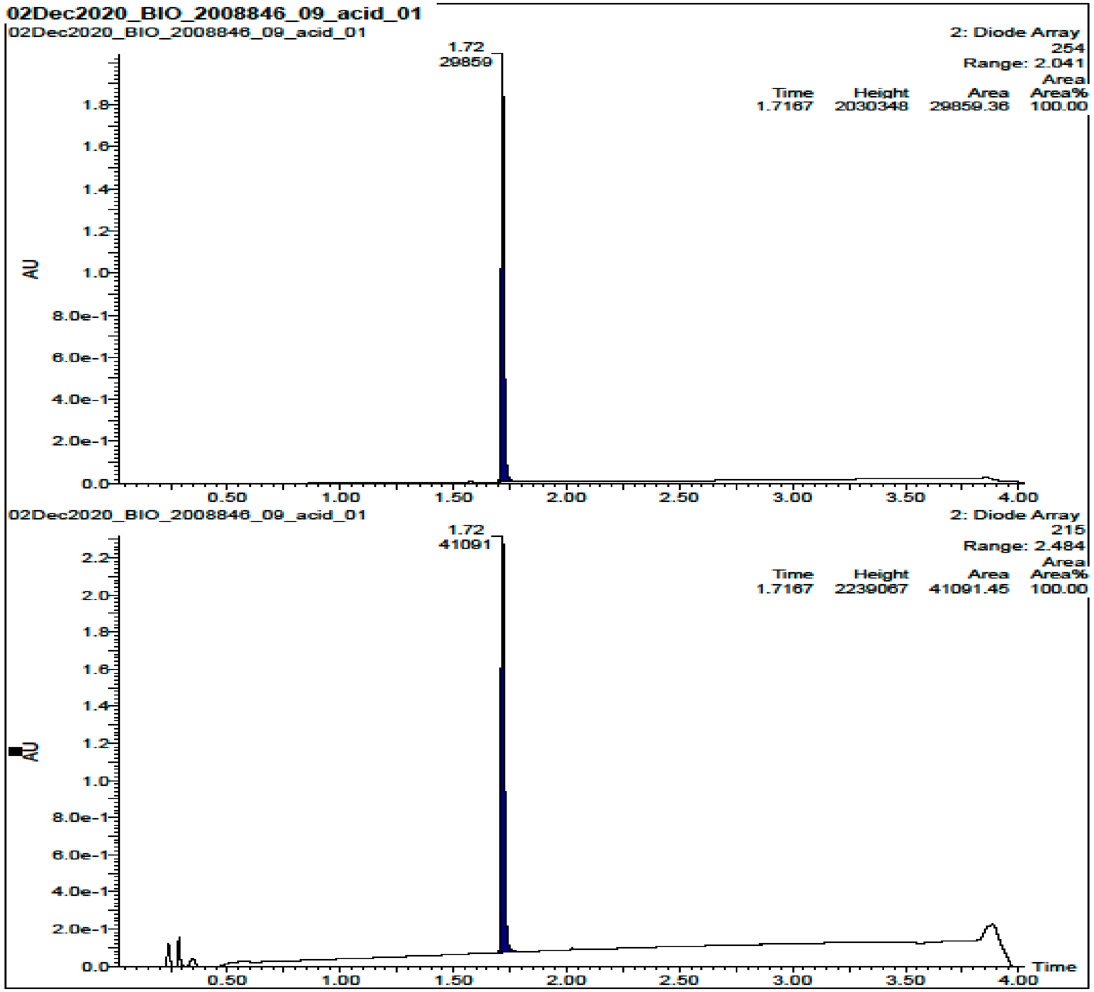

HPLC–purity, basic conditions:

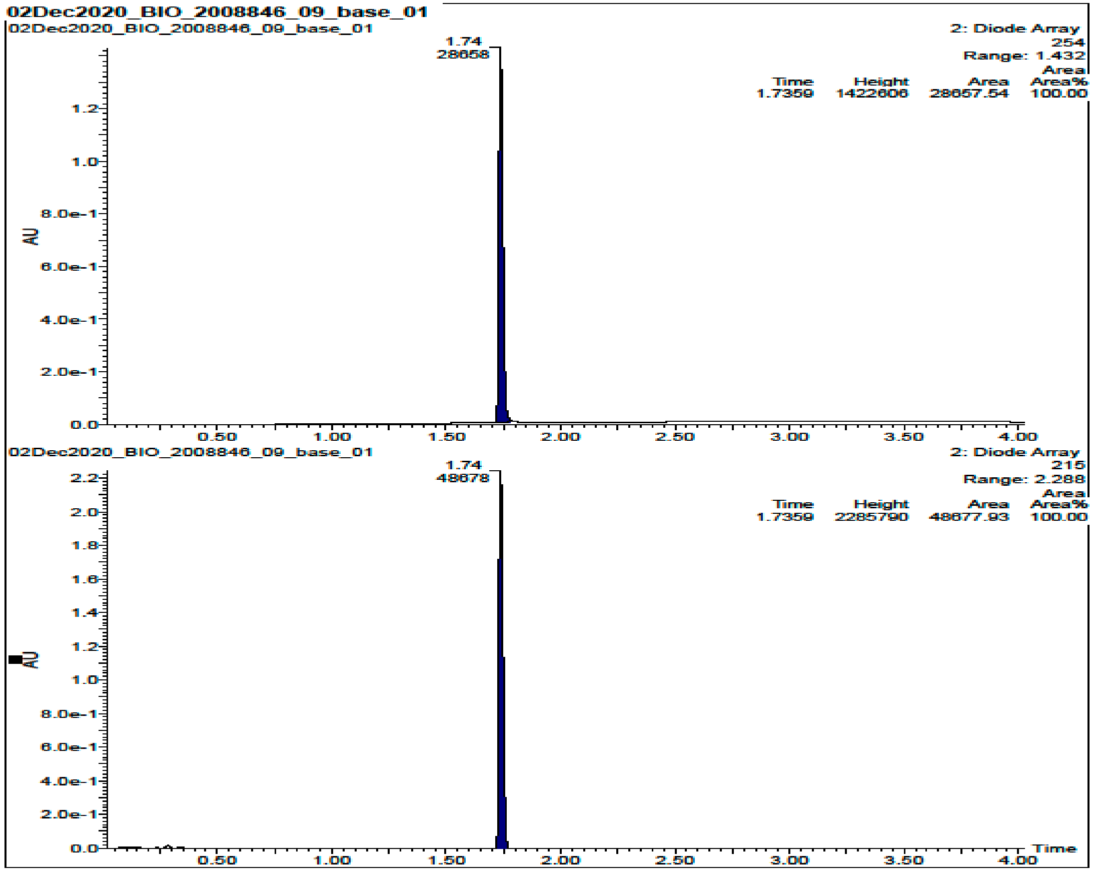

DSC; melting point:

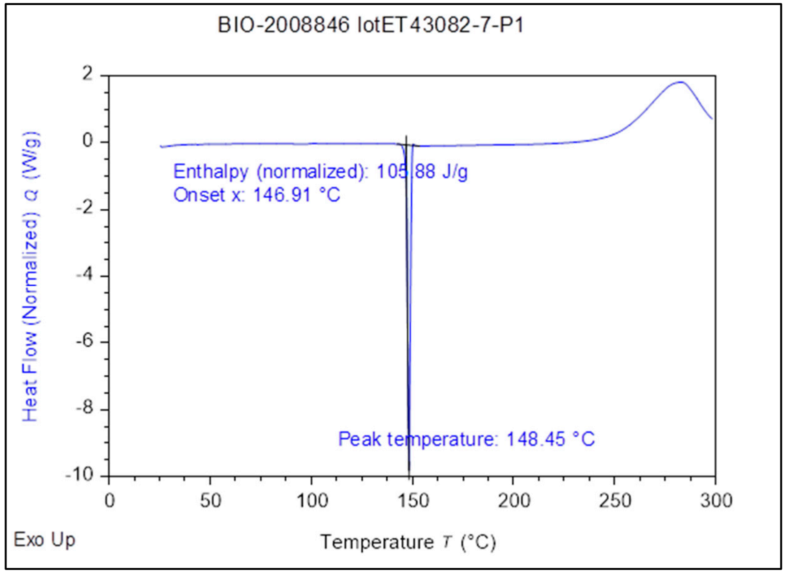

BIO-2011057

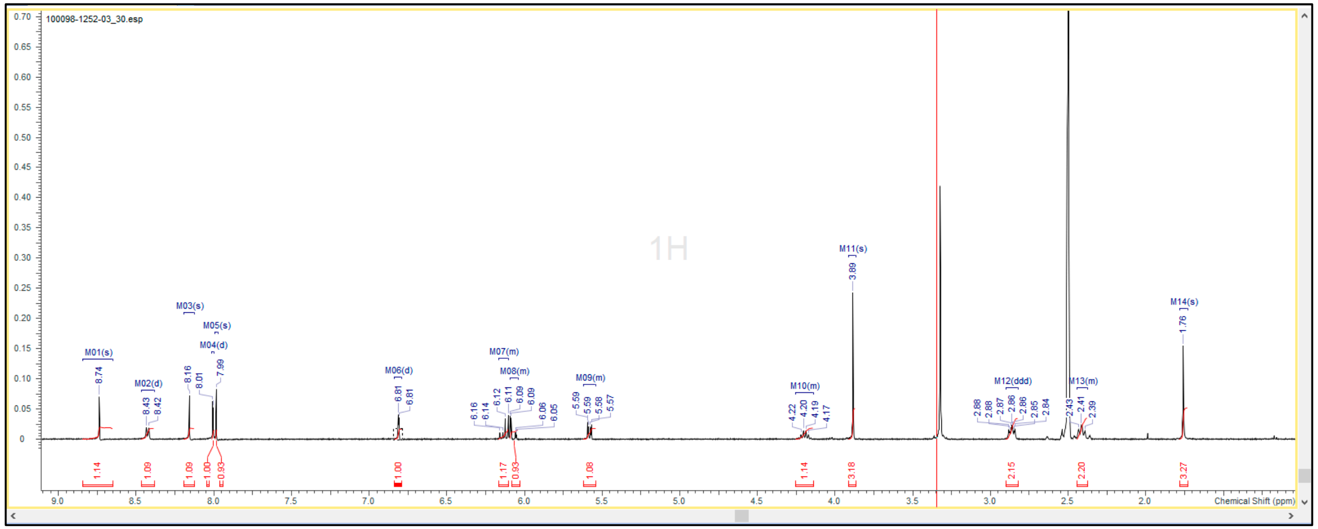

^{1}H NMR (500 MHz, DMSO-d_6) δ ppm 1.76 (s, 3 H) 2.38 - 2.44 (m, 2 H) 2.86 (ddd, *J*=9.92, 7.17, 3.05 Hz, 2 H) 3.89 (s, 3 H) 4.14 - 4.25 (m, 1 H) 5.54 - 5.62 (m, 1 H) 6.04 - 6.09 (m, 1 H) 6.10 - 6.17 (m, 1 H) 6.81 (d, *J*=2.44 Hz, 1 H) 7.99 (s, 1 H) 8.00 (d, *J*=2.44 Hz, 1 H) 8.16 (s, 1 H) 8.43 (d, *J*=7.94 Hz, 1 H) 8.74 (s, 1 H)

HPLC–purity (base modifier):

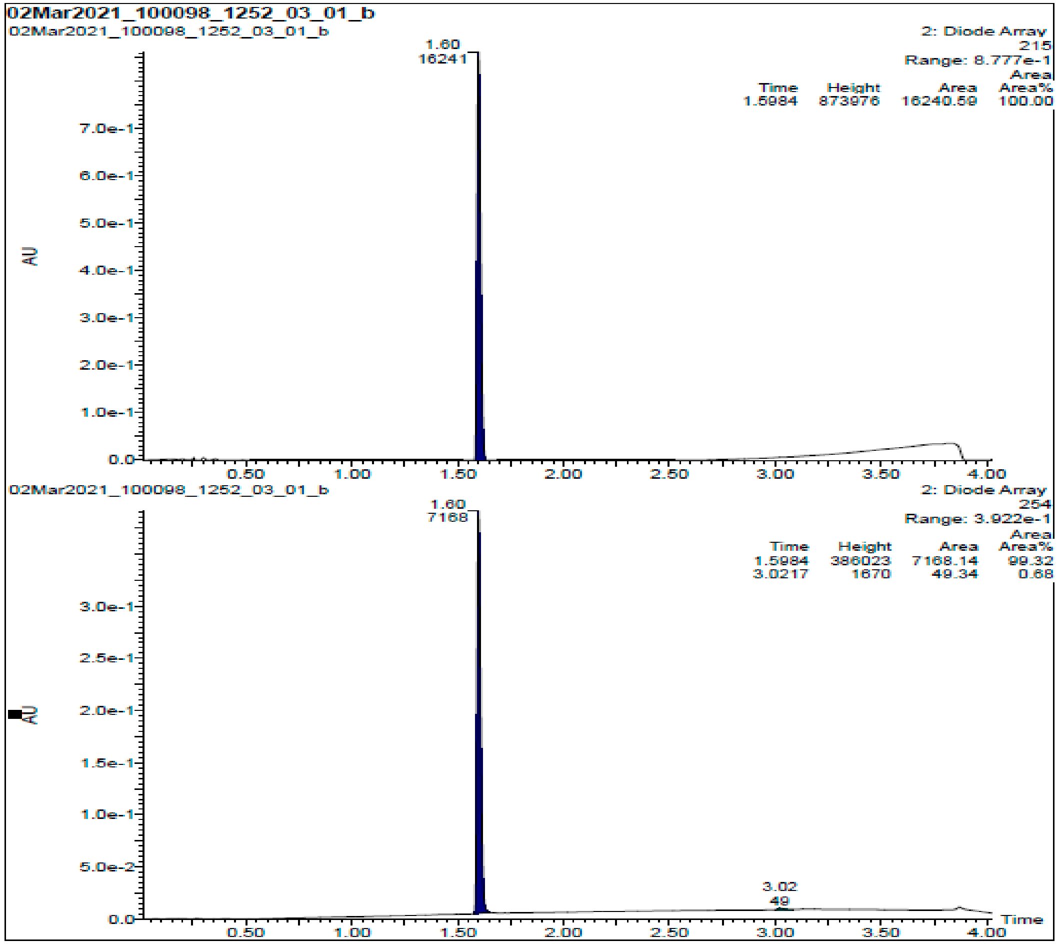

HPLC–purity (acid modifier):

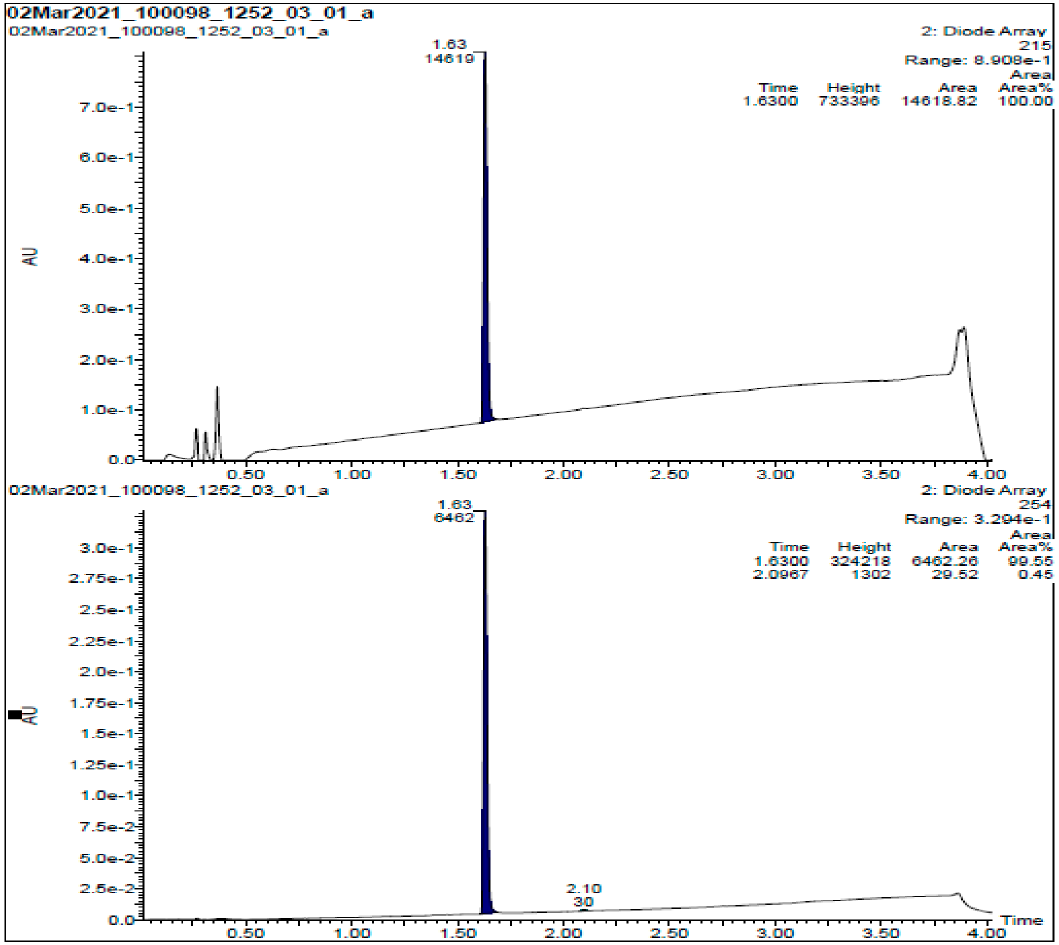

Optical purity:

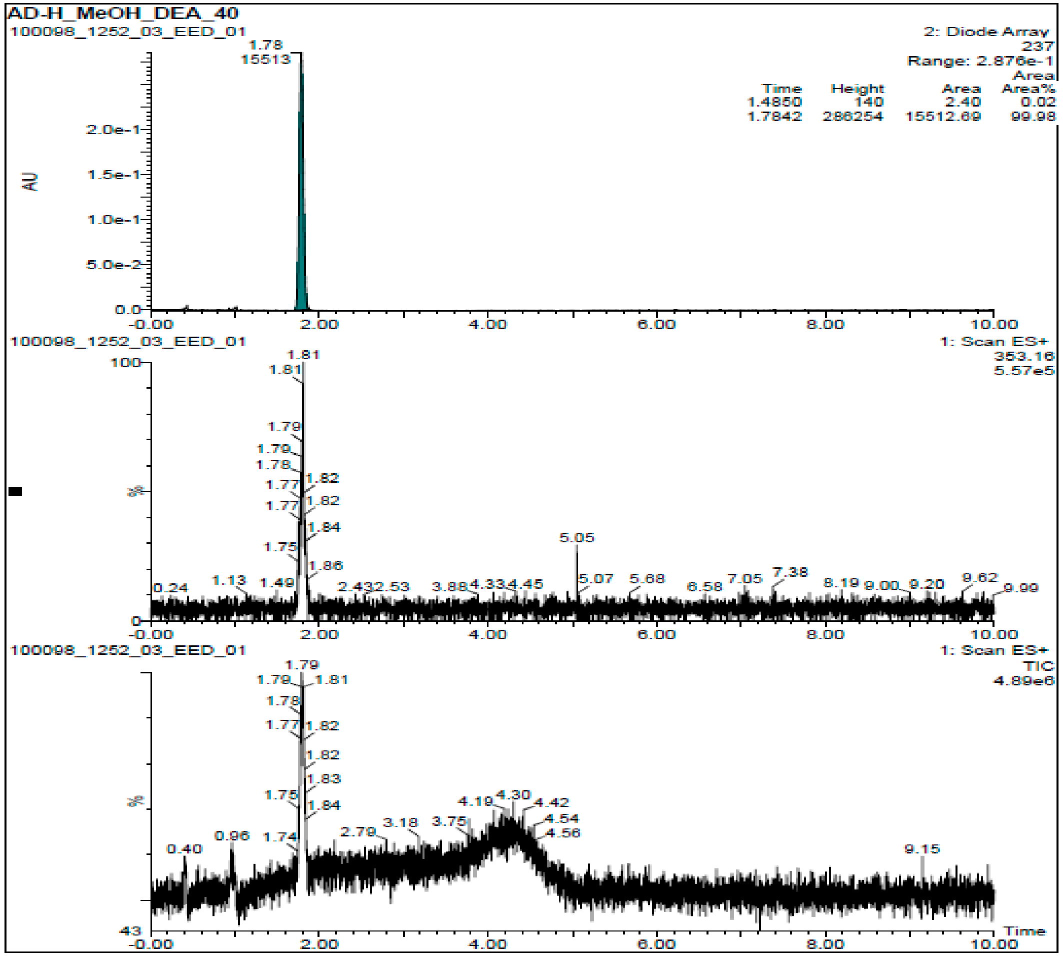

BIO-2012384

^{1}H-NMR:

^{1}H-NMR:

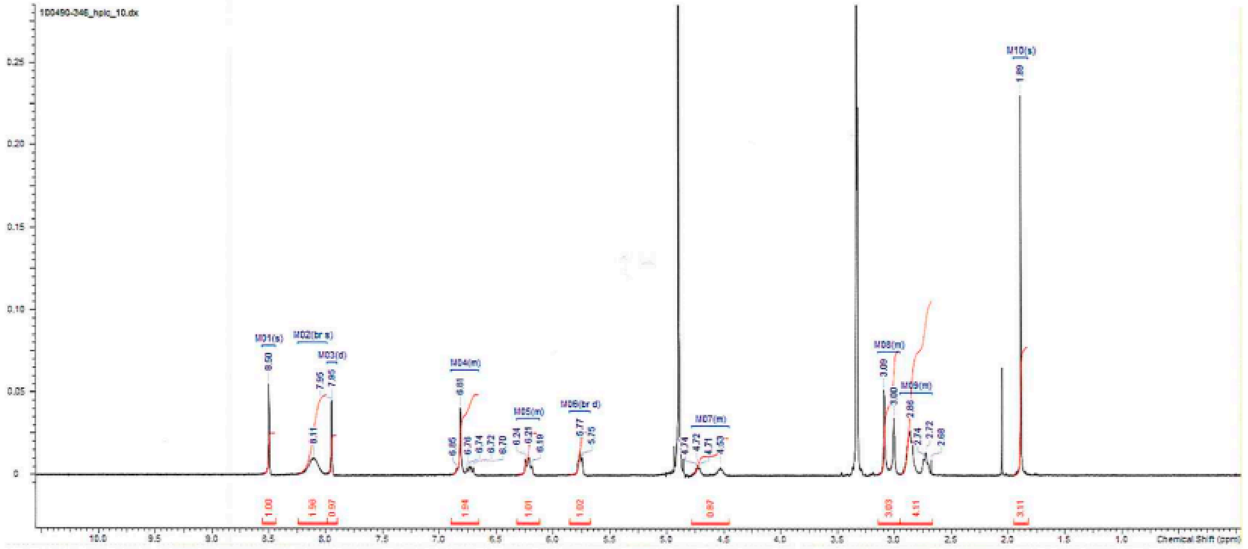

^{1}H NMR (500 MHz, METHANOL-d_4) δ 8.50 (s, 1H), 8.11 (br s, 2H), 7.95 (d, *J*=2.44 Hz, 1H), 6.65-6.90 (m, 2H), 6.12-6.32 (m, 1H), 5.76 (br d, *J*=9.77 Hz, 1H), 4.45-4.78 (m, 1H), 2.95-3.14 (m, 3H), 2.66-2.95 (m, 4H), 1.89 (s, 3H)

HPLC–purity, acidic conditions:

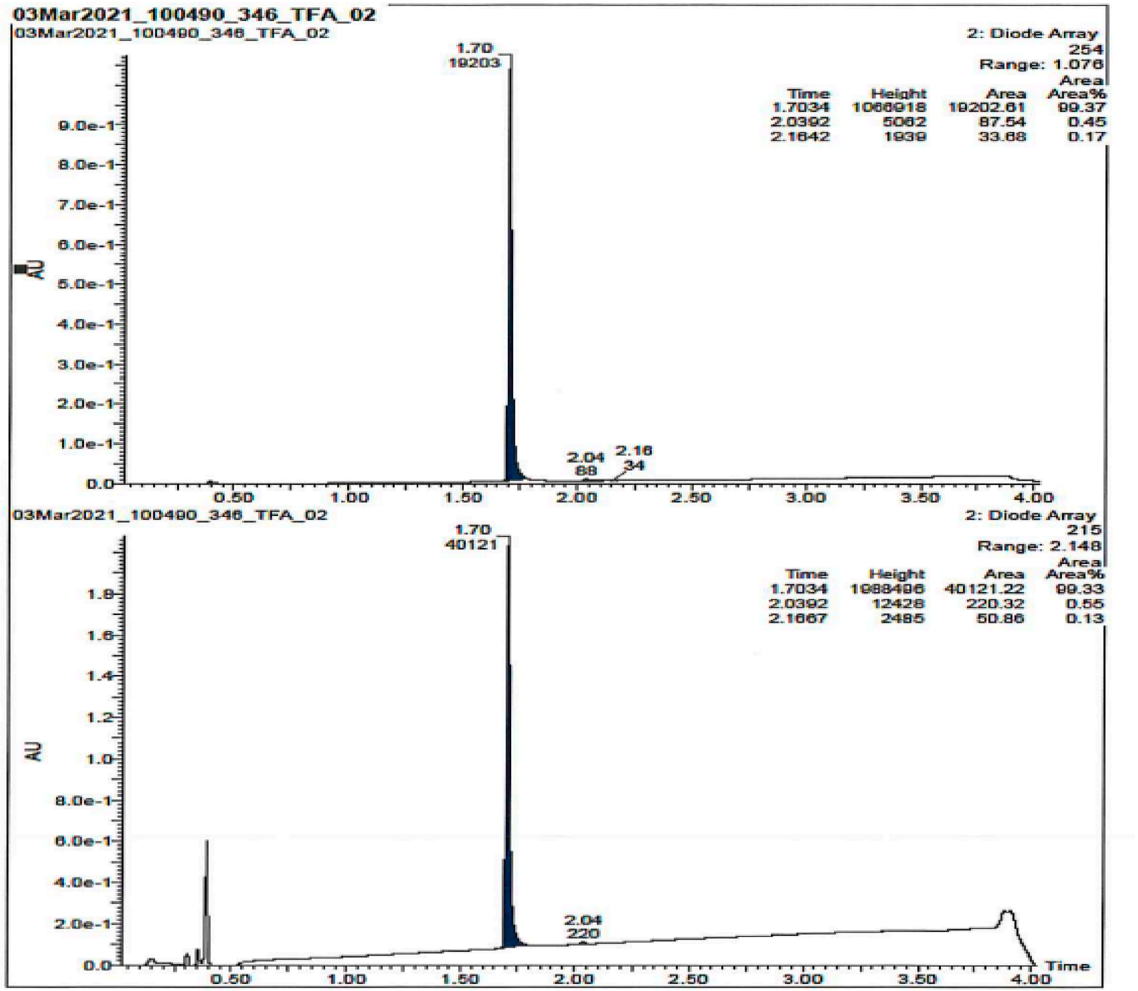

HPLC–purity, basic conditions:

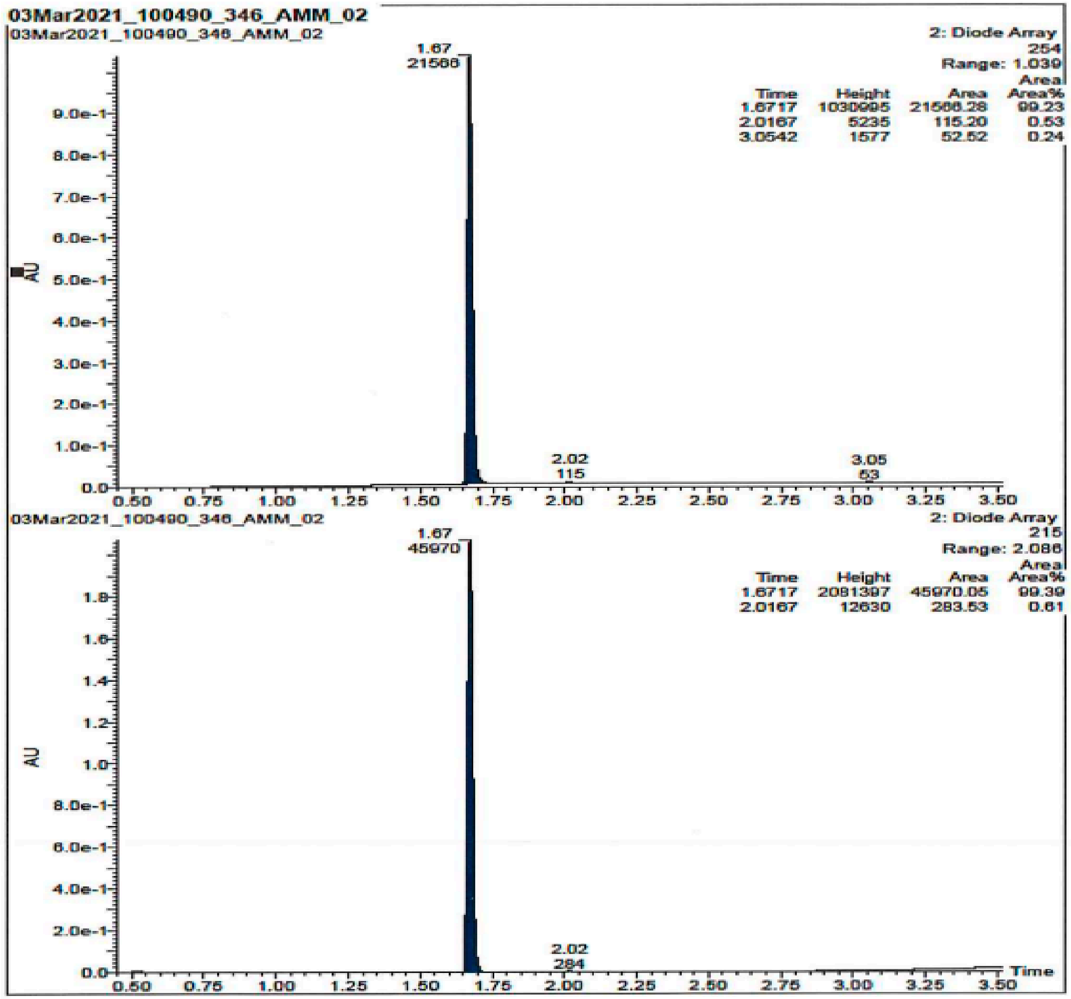

BIO-2059886

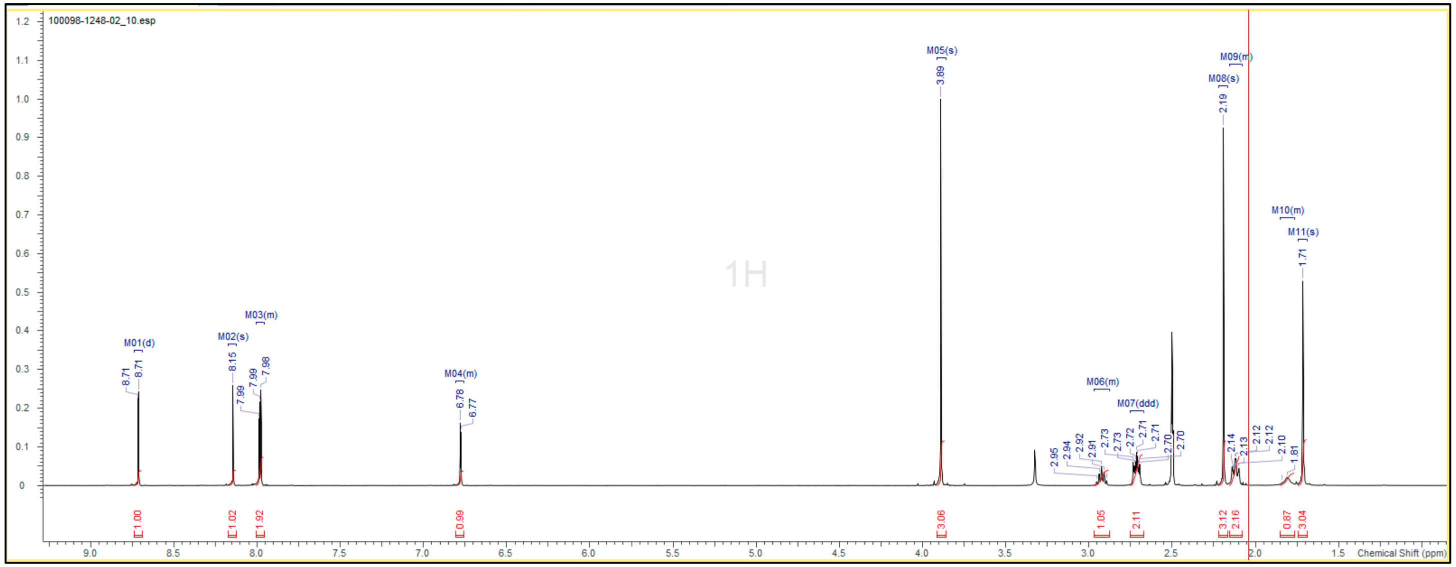

^{1}H NMR (500 MHz, DMSO-d_6) δ ppm 1.71 (s, 3 H) 1.76 - 1.85 (m, 1 H) 2.08 - 2.16 (m, 2 H) 2.19 (s, 3 H) 2.71 (ddd, *J*=9.31, 6.56, 3.05 Hz, 2 H) 2.88 - 2.97 (m, 1 H) 3.89 (s, 3 H) 6.76 - 6.80 (m, 1 H) 7.96 - 8.01 (m, 2 H) 8.15 (s, 1 H) 8.71 (d, *J*=1.22 Hz, 1 H)

HPLC–purity (base modifier):

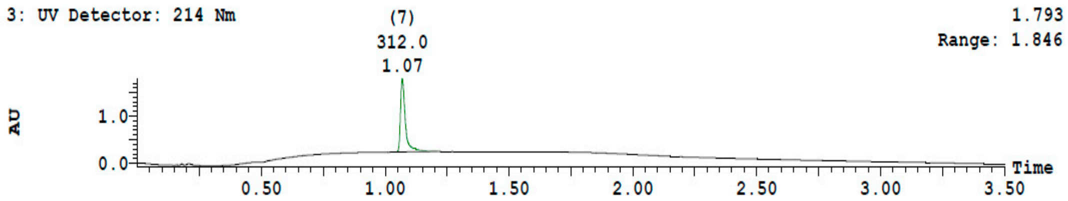

Peak Number	Time	Area %Total	Mass Found
7	1.07	100.00	312.0000

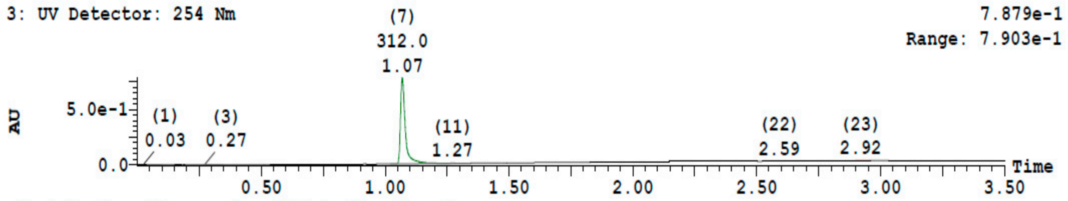

Peak Number	Time	Area %Total	Mass Found
1	0.03	0.89	
3	0.27	0.50	
7	1.07	96.75	312.0000
11	1.27	0.60	
22	2.59	0.71	Not Found
23	2.92	0.56	Not Found

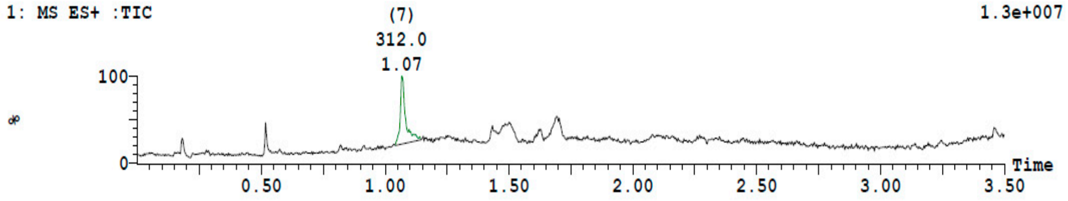

HPLC–purity (acid modifier):

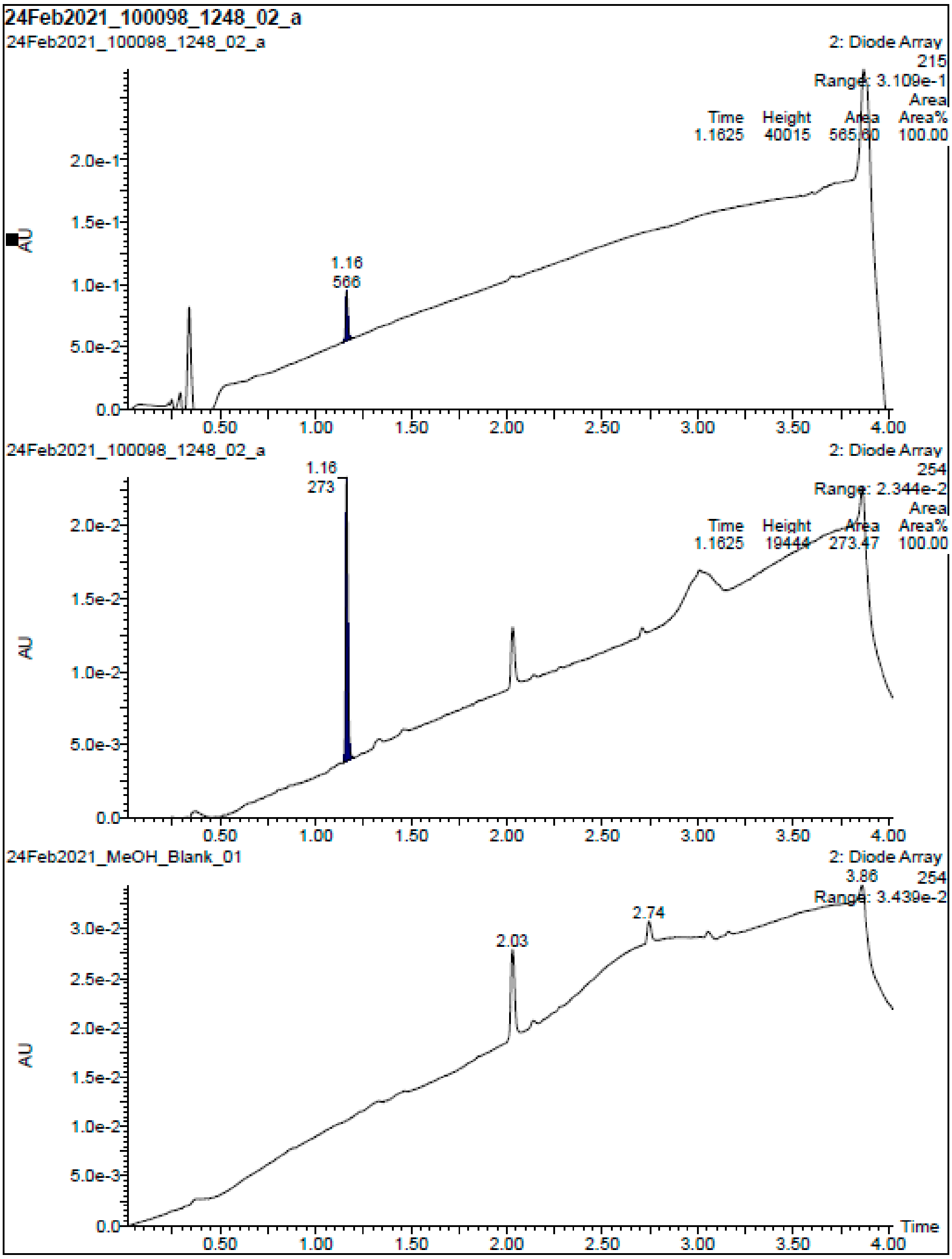

Optical purity:

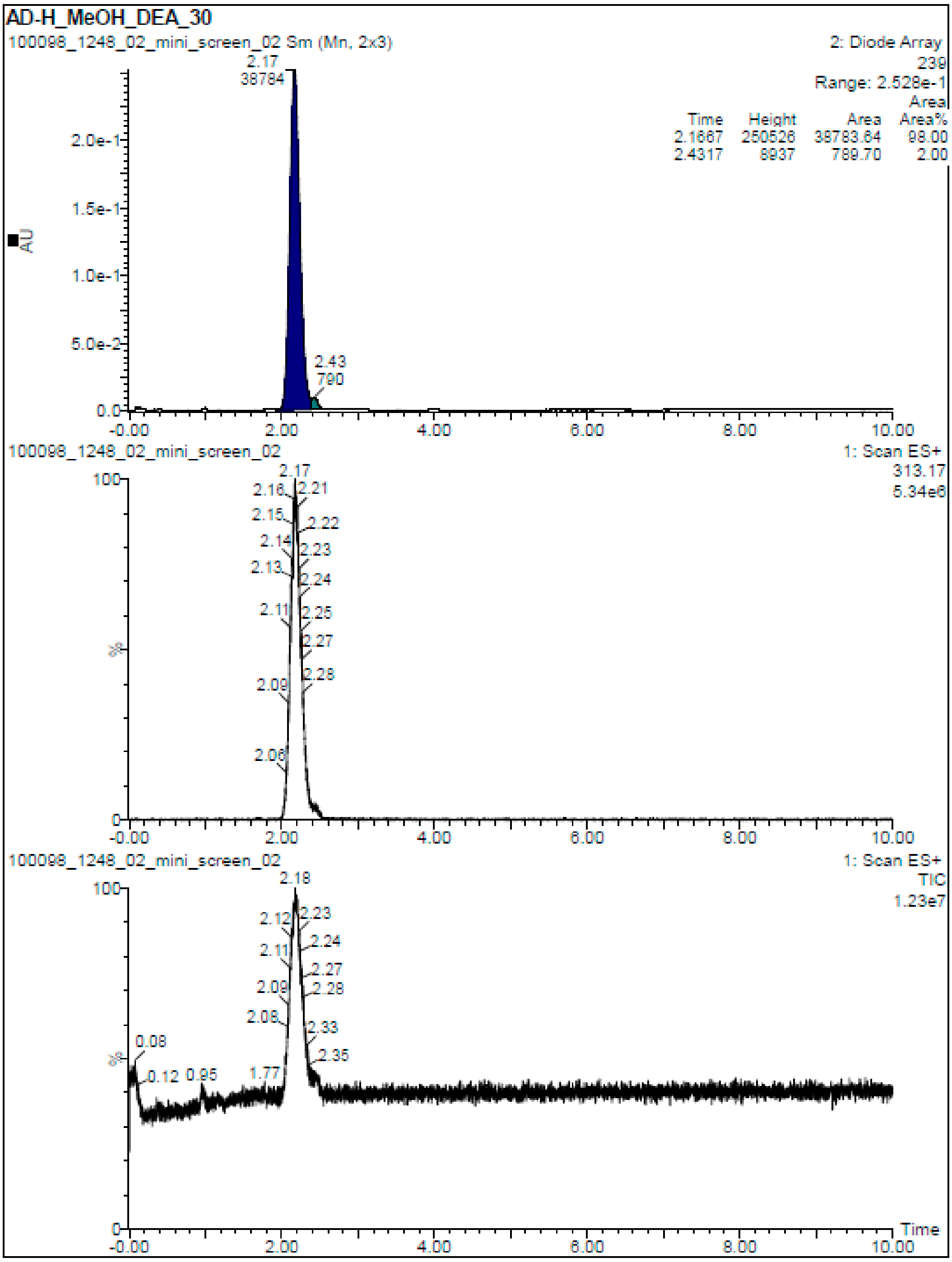
AD-H_MeOH_DEA_30
100098_1248_02_mini_screen_02 Sm (Mn, 2x3)
2: Diode Array
239
Range: 2.528e-1
Time Height Area Area%
2.1667 250526 38783.64 98.00
2.4317 8937 789.70 2.00
2.17
38784
2.43
790
AU
2.0e-1
1.5e-1
1.0e-1
5.0e-2
0.0
-0.00
2.00
4.00
6.00
8.00
10.00
100098_1248_02_mini_screen_02
1: Scan ES+
313.17
5.34e6
2.17
2.16
2.21
2.15
2.22
2.14
2.23
2.13
2.24
2.11
2.25
2.27
2.09
2.28
2.06
%
100
0
100098_1248_02_mini_screen_02
1: Scan ES+
TIC
1.23e7
2.18
2.12
2.23
2.11
2.24
2.27
2.09
2.28
2.08
2.33
0.08
2.35
0.12
0.95
1.77
Time

S2: TACs for whole brain from all four PET measurements were assessed using kinetic modelling both two-tissue compartment model (2TCM) and graphical analyses using Ichise Multilinear Analysis MA1 (MA1).

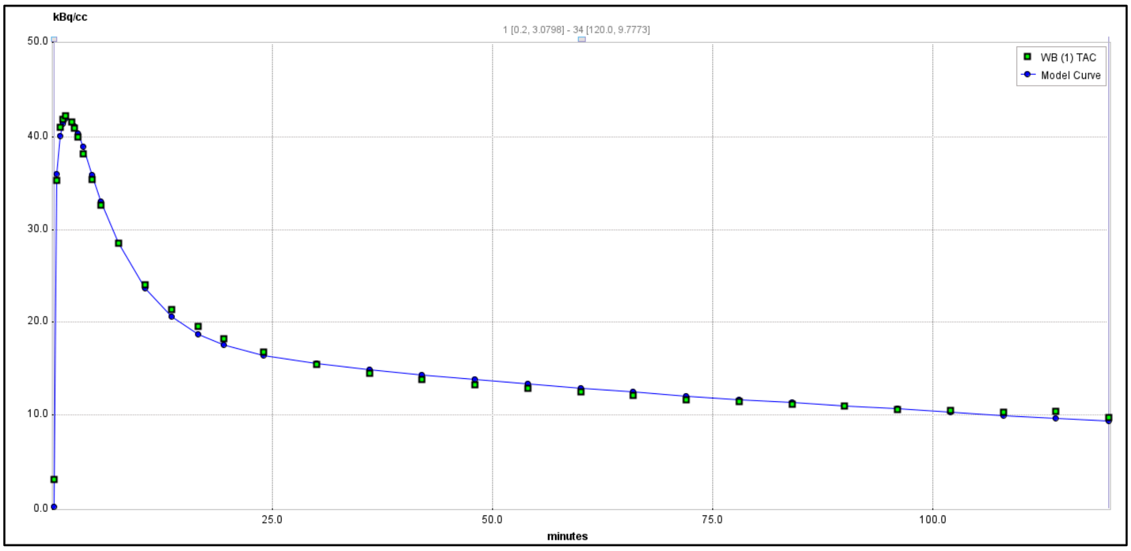

The whole brain time-activity curve (TAC) for PET measurements on NHP1 under baseline conditions was analyzed using the two-tissue compartment model (2TCM) for kinetic modeling.

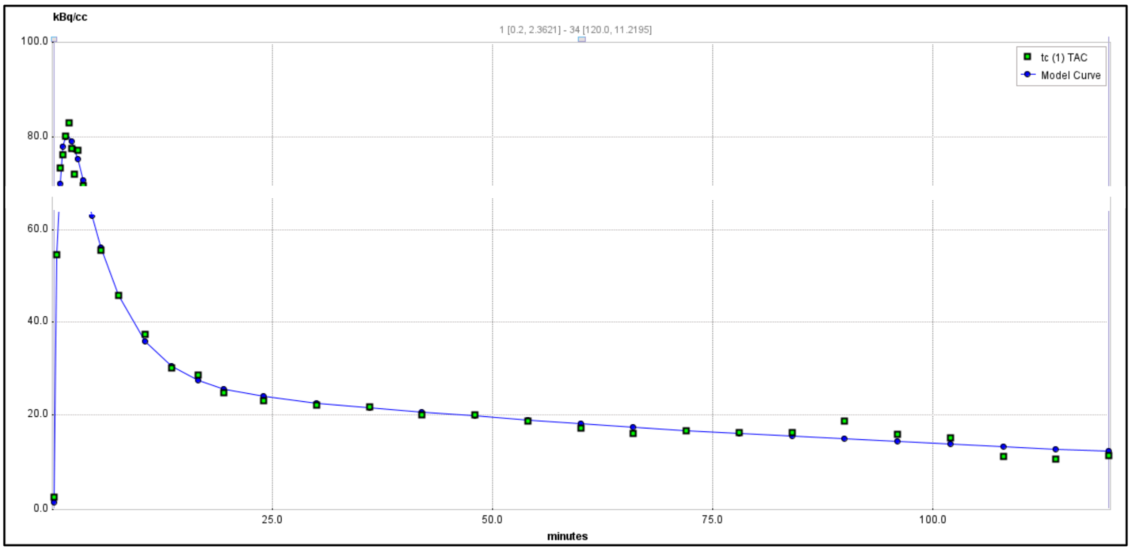

The whole brain time-activity curve (TAC) for PET measurements on NHP1 after pretreatment conditions was analyzed using the two-tissue compartment model (2TCM) for kinetic modeling.

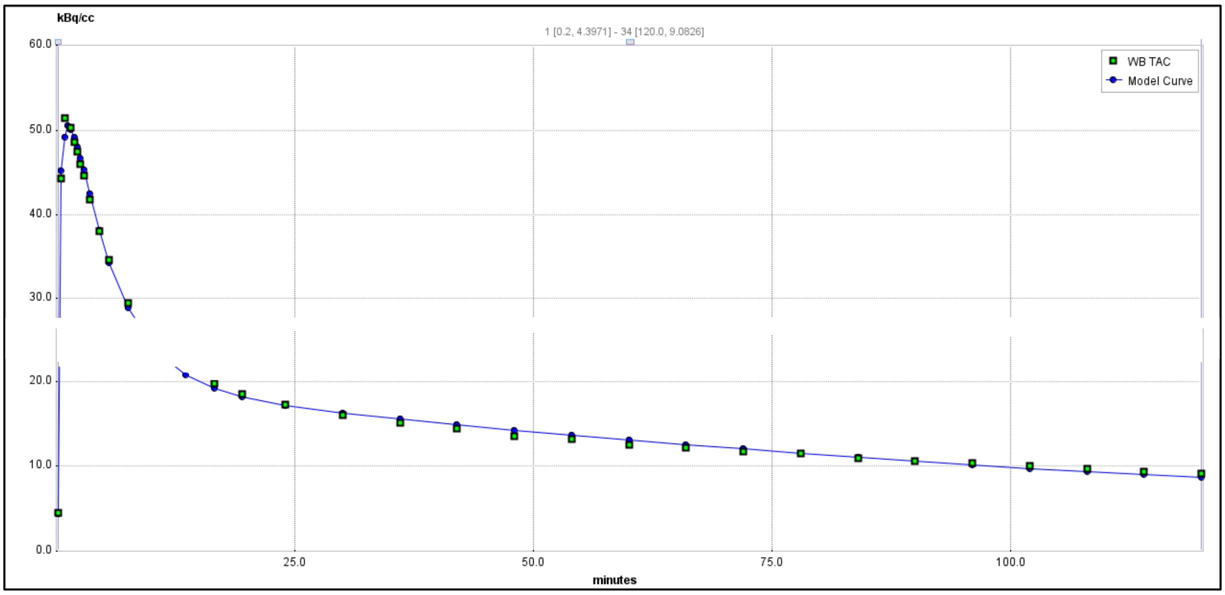

The whole brain time-activity curve (TAC) for PET measurements on NHP1 under baseline conditions was analyzed using the two-tissue compartment model (2TCM) for kinetic modeling.

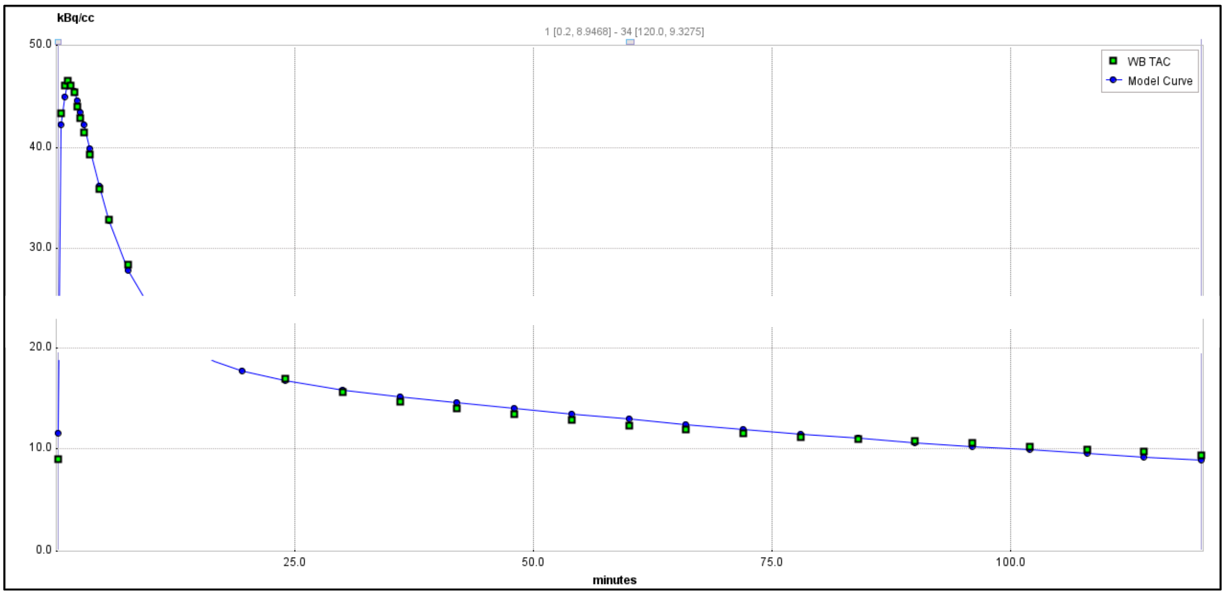

The whole brain time-activity curve (TAC) for PET measurements on NHP2 after pretreatment conditions was analyzed using the two-tissue compartment model (2TCM) for kinetic modeling.

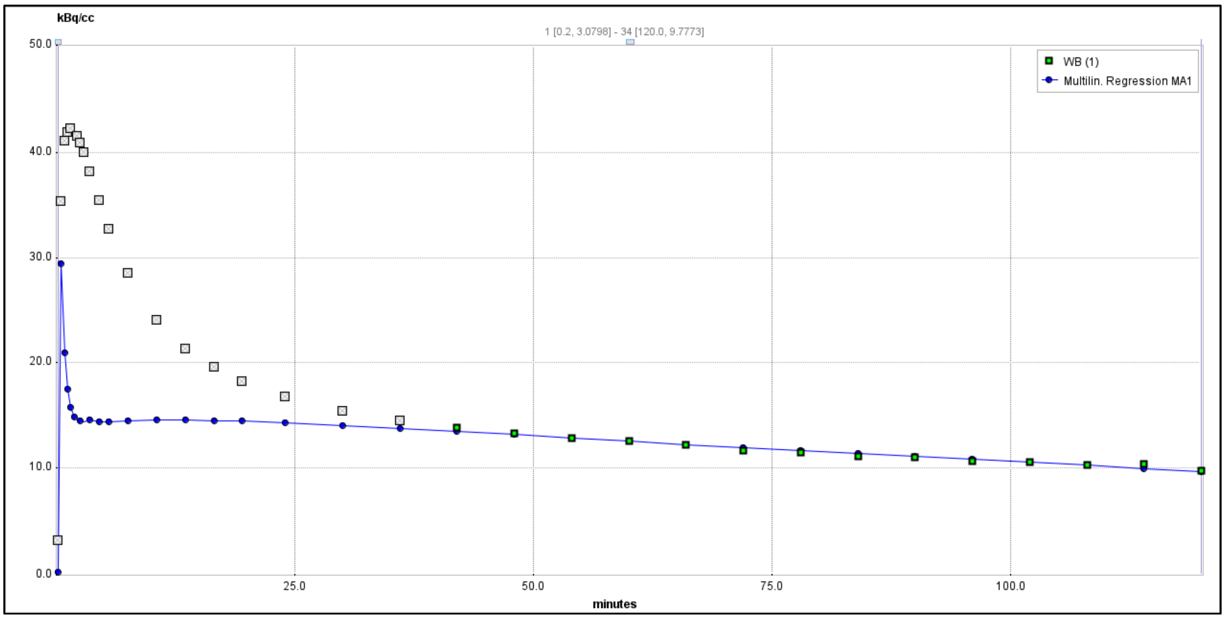

The whole brain time-activity curve (TAC) for PET measurements on NHP1 under baseline conditions was analyzed using the Multilinear Analysis MA1

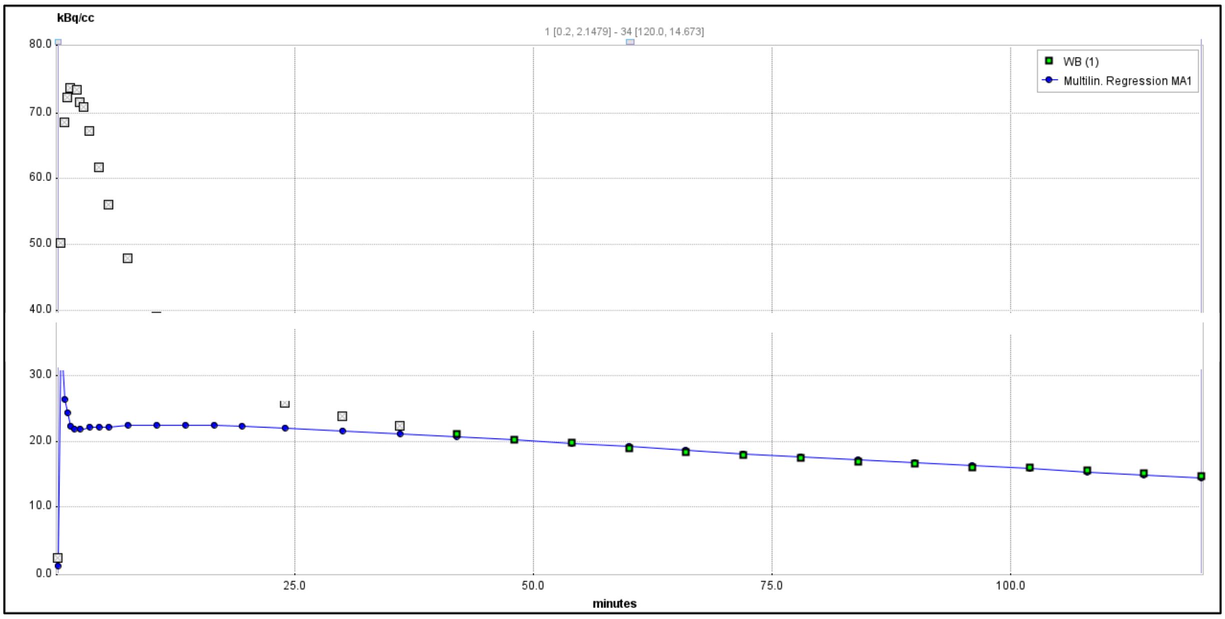

The whole brain time-activity curve (TAC) for PET measurements on NHP1 after pretreatment conditions was analyzed using the Multilinear Analysis MA1

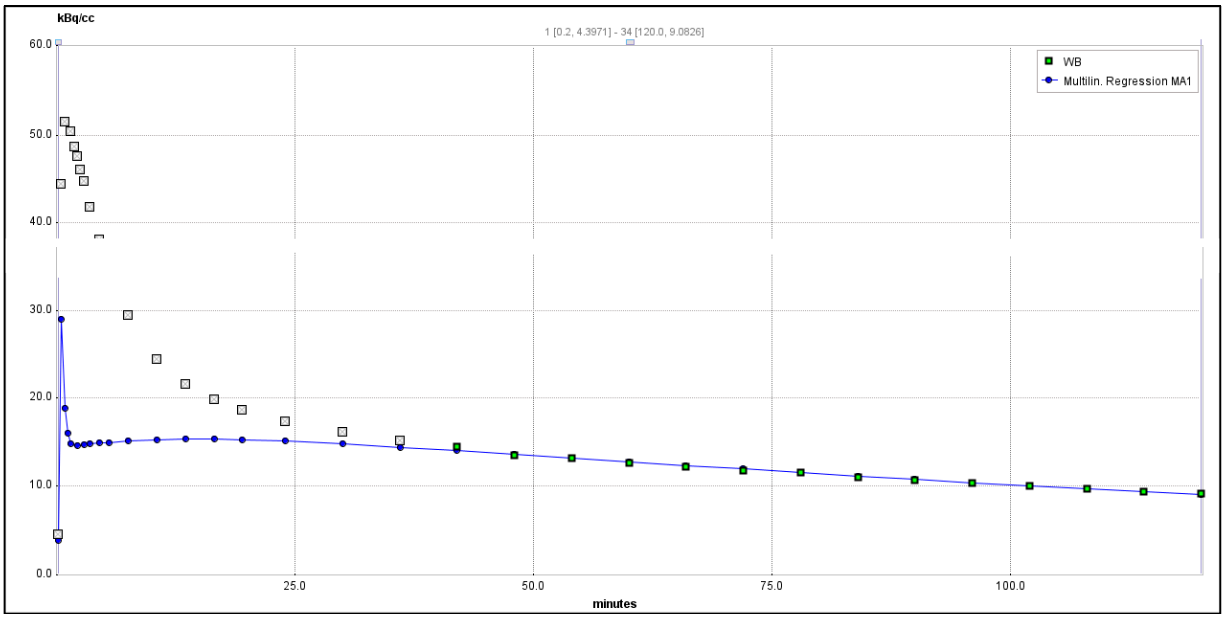

The whole brain time-activity curve (TAC) for PET measurements on NHP2 under baseline conditions was analyzed using the Multilinear Analysis MA1

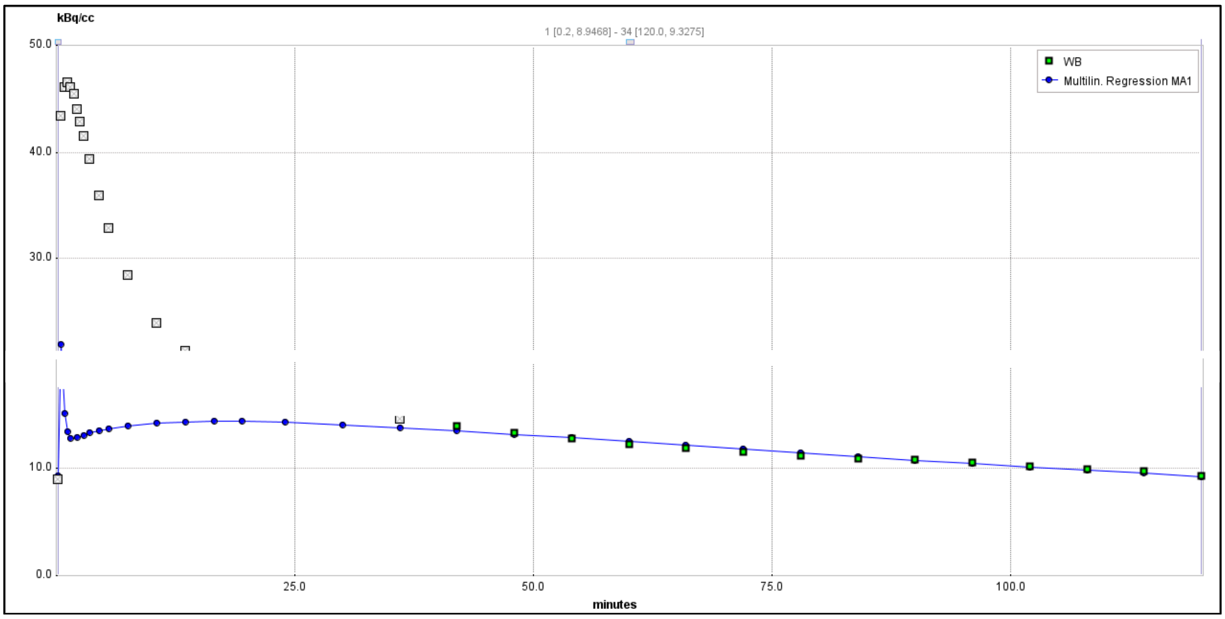

The whole brain time-activity curve (TAC) for PET measurements on NHP2 after pretreatment conditions was analyzed using the Multilinear Analysis MA1
